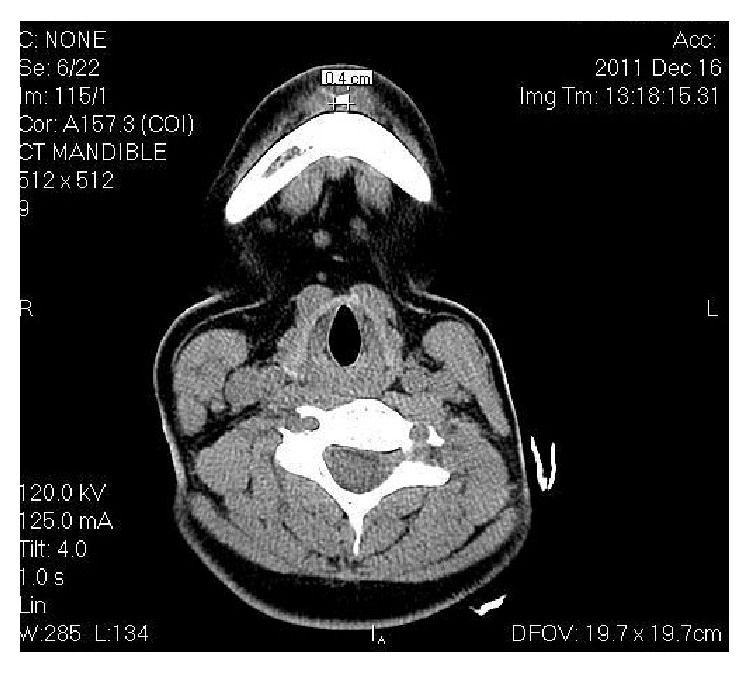# Orocutaneous Fistula or Traumatic Infectious Skin Lesion: A Diagnostic Dilemma

**DOI:** 10.1155/2015/353069

**Published:** 2015-11-04

**Authors:** Mayank Vermani, Vimal Kalia, Sumita Singh, Sunny Garg, Shweta Aggarwal, Richa Khurana, Geeta Kalra

**Affiliations:** ^1^Pt. BD Sharma University of Health Sciences, Rohtak 124001, India; ^2^Department of Oral and Maxillofacial Surgery, JCD Dental College, Sirsa, Haryana 125055, India; ^3^Department of Oral and Maxillofacial Surgery, BRS Dental College, Sultanpur, Panchkula 134118, India; ^4^Department of Oral and Maxillofacial Surgery, Punjab Institute of Medical Sciences, Jalandhar 144022, India; ^5^Private Practice, Maur Mandi, Bathinda 151509, India; ^6^Department of Periodontics, JCD Dental College, Sirsa, Haryana 125055, India

## Abstract

Orocutaneous fistula (OCF) (of dental origin) is an uncommon but well-described condition in the literature. These are often misdiagnosed by physicians and dentists. Careful selection of investigating modality is important in case of diagnostically challenging cases. A 19-year-old female came with a complaint of a lesion on the chin reported with h/o trauma with the impact on chin presented as diagnostic dilemma because of unusual case history and clinical examination. Commonly used radiographic investigations like IOPA and orthopantomograph did not resolve the dilemma whereas advanced imaging modality like CT scan, 3D volume imaging, and contrast enhanced CT played an important role in the diagnosis of OCF and selecting the treatment plan.

## 1. Introduction

Orocutaneous fistula (OCF) (of dental origin) is an uncommon but well-described condition in the literature. Diagnosis of OCF is still challenging and should be done with careful history taking, clinical examination, and radiographic examination. Such conditions are often misdiagnosed by physicians and dentists as skin lesions or nonodontogenic lesion. Therefore, accurate diagnosis and use of correct investigating modality are important for the diagnosis of OCF (of dental origin) for prompt treatment and reducing complication such as asepsis, osteomyelitis, and patient discomfort. This case describes the patient with lesion on the chin presented as diagnostic dilemma because of unusual history and clinical presentation which resulted in confusion whether the lesion is because of dental origin or a separate entity. This case report also describes the importance of CT scan, 3D volume imaging, and contrast enhanced CT in the diagnosis of OCF and in visualizing the communication between the periapical region and the extraoral lesion. This imaging technique might be very helpful to improve the diagnosis and management of orocutaneous fistula.

## 2. Case Report

A healthy 19-year-old female came to the department of oral and maxillofacial surgery with a chief complaint of pus oozing from small opening above the chin since past 1 month. Past medical history revealed h/o trauma by falling 4 yrs back. Since then the extraoral lesion was present. Patient was unable to bite from lower front teeth since 1 month. Pain was dull, continuous, and nonradiating, which aggravates on mastication and relieves on taking analgesics.

**Figure 1:**
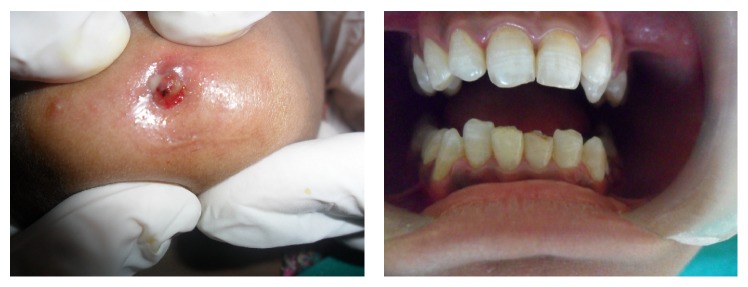


The patient stated that the lesion had been a frequent discharge of purulent material (Figure 1), but the symptoms decreased when she used antibiotics. However, the pus recurred every time she stopped “therapy.” Clinical examination revealed that all mandibular anterior teeth have normal appearance concerning integrity of their crowns whereas occlusal tips of lower incisors were attrisoned or could be an ellis class I fracture that happened during trauma 4 months back. Pulp sensitivity tests responded within normal limits.

On radiographic examination, IOPA (Figure 3) was taken with GP point inserted into lesion which revealed a periapical radiolucency involving right and left mandibular central incisor that may or may not involve other teeth. It is a blind procedure and can result in misdiagnosis because of overlapping. OPG also showed periapical radiolucency (6.8 × 0.4 mm) associated with right central, left central, and left lateral incisor but does not give any information regarding fistulous tract. Being 2-dimensional OPG (Figure 4) did not give any additional information regarding pathology and resulted in a diagnostic dilemma. Despite the extensive apical and lateral areas of radicular involvement, mimicking the so-called “endo-perio” lesion, the teeth were firm (no signs of mobility) and did not reveal any periodontal pockets on probing.

On CT scan (Figure 5), involvement of all 4 anterior teeth, that is, right and left mandibular central and lateral incisors, can be appreciated. CT also described the exact size (0.7 × 1 cms) and extent of the pathology. 3D volume imaging (Figure 6) showed the size and path of fistulous tract and delineated the pathology from normal anatomy. Further, with the help of contrast enhanced CT scan (CECT) imaging (Figure 7) helped in visualizing the course of fistula (0.4 cms) with the help of contrast media which confirmed the diagnosis. CECT also helped in formulating a treatment plan.

## 3. Discussion

**Figure 2:**
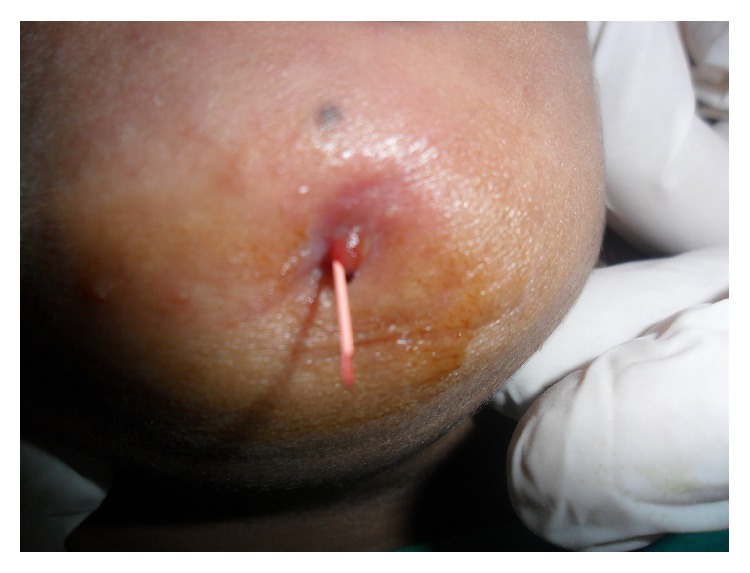


**Figure 3:**
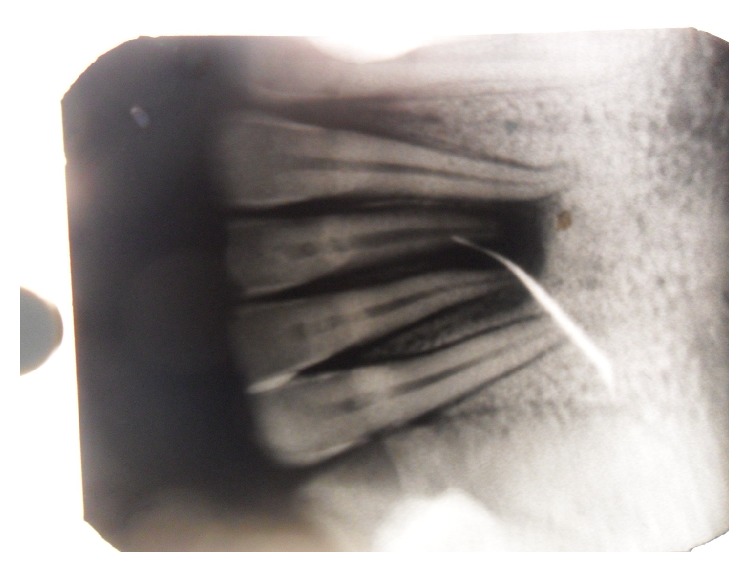


**Figure 4:**
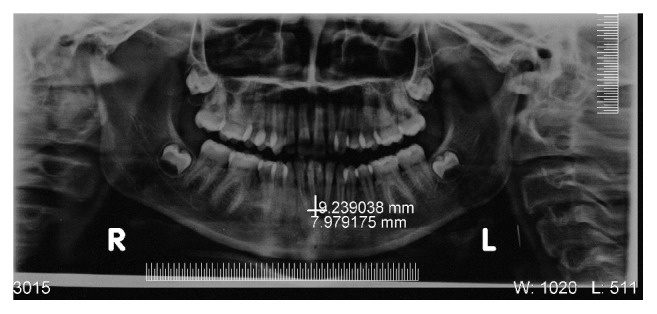


**Figure 5:**
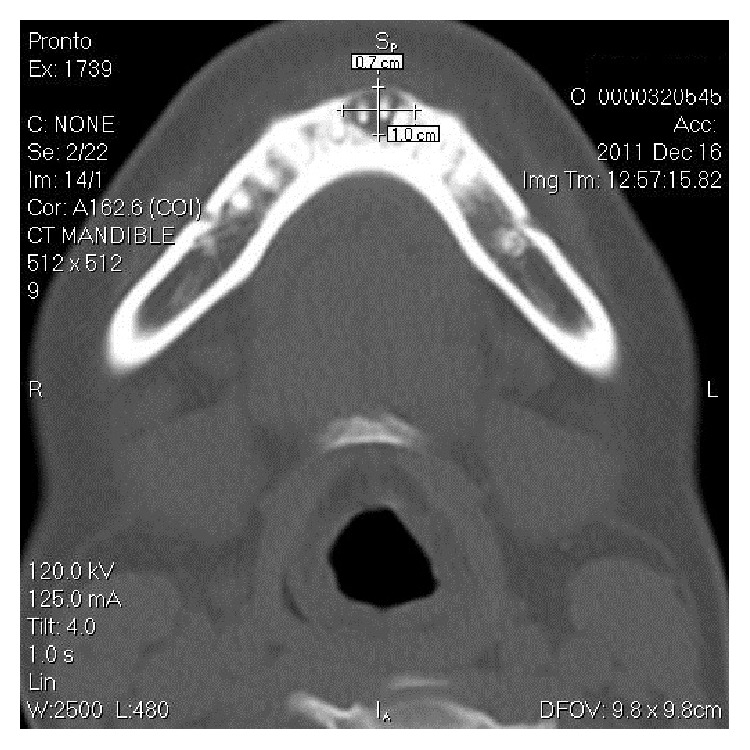


Term orocutaneous fistula is misleading especially if it is of dental origin because there is no direct contact of oral cavity and skin. We suggest the use of terms like odontocutaneous, dentocutaneous, or apicocutaneous fistula for OCF of dental origin. An extra oral opening or cutaneous sinus tract may be confused with wide variety of diseases including local skin infections, in grow hair or occluding sweat gland duct, osteomyelitis, neoplasm, tuberculosis, actinomycosis, congenital midline sinus of upper lip [1–8], congenital fistula and infected cyst, pyogenic granuloma, and other pathologies [9]. Treatment varies for different pathologies; therefore, accurate diagnosis and understanding of the lesion are important. OCF of dental origin develops from dental abscess or after trauma. The areas most commonly affected are chin and submental regions [10]. It can be investigated by testing pulp vitality, by tracking the sinus tract to its origin with gutta percha, and by radiographic examination. Testing the pulp vitality determines the condition of tooth but does not describe the path of fistula. Also insertion of GP point (Figures 2 and 3) may describe the direction and depth of fistula, presence of foreign body, and communication of fistula with apices of mandibular incisors and fresh discharge came out on withdrawal, but it is a blind procedure and 2D imaging with IOPA or orthopantomogram may result in false diagnosis because of overlapping.

**Figure 6:**
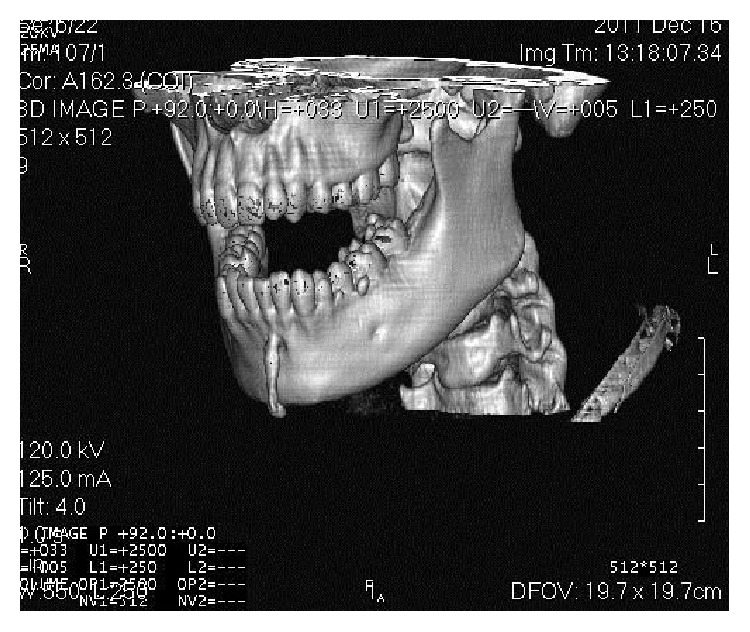


Radiological examination plays an important role in diagnosis of OCF. IOPA and OPG are the frequently ordered radiographs for the diagnosis of OCF. Odontogenic cutaneous sinus tract on the face often develops as a result of chronic apical periodontitis caused by pulpal degeneration or necrosis [11]. Periapical infection spreads through medullary bone and perforates cortical bone and enters soft tissue. In soft tissue it follows the path of least resistance and perforates on mucosal or extraoral cutaneous surface, for which commonly used imaging modalities like IOPA and OPG are not enough. They show the 2-dimensional view of 3-dimensional structures. Film elongation and shortening, geometric distortions, and being unable to describe the fistulous tract are other disadvantages of these radiographs. Transcortical extension of the inflammatory process can result in cortical destruction, fistulization, and periosteal reaction, and all these changes can be evaluated very well by CT [12]; thus, advanced diagnostic modalities like CT scan, 3-dimensional volume imaging, and CECT are required which visualise the topography and morphology of pathology in hard as well as soft tissues. Correct CT examination enables an accurate and timely diagnosis, which provides valuable indications for treatment. CT imaging displays bony details and gives exact information about the size, extent, origin, content, and relationships of the lesion with vital structures in the mandible. 3D volume imaging (Figure 6) delineated the pathology from normal anatomy and CT with iodinated contrast media revealed the path of fistulous tract, that is, 0.4 cms (Figure 7). That is why, this imaging technique should be preferred to CT without media contrast, which only shows indirect signs of orocutaneous fistula. Hence, CECT is an essentiality for the diagnosis of OCF and should be ordered by the clinicians in order to reach the accurate diagnosis and execute the best treatment plan.

## 4. Conclusion

A case of OCF is reported which presented as a diagnostic dilemma which was resolved with the help of advanced radiographic investigations. All orocutaneous fistula must be considered of dental origin. The diagnosis of orocutaneous fistula must be as quick as possible to improve the clinical outcome. Therefore, early and accurate diagnosis demands that the lesion should be investigated with advanced imaging modalities like CT scan, 3-dimensional volume imaging, and CECT. Hence, in case of confusion, a clinician should ask for advanced imaging modalities like CT scan, 3-dimensional volume imaging, and CECT rather than depending upon imagination and conceptualization of the pathology.

## Figures and Tables

**Figure 7:**